# Conversion of acetate and glyoxylate to fumarate by a cell-free synthetic enzymatic biosystem

**DOI:** 10.1016/j.synbio.2023.03.004

**Published:** 2023-03-16

**Authors:** Congli Hou, Linyue Tian, Guoli Lian, Li-Hai Fan, Zheng-Jun Li

**Affiliations:** a College of Chemical Engineering, Fujian Engineering Research Center of Advanced Manufacturing Technology for Fine Chemicals, Fuzhou University, Fuzhou, 350108, People's Republic of China; b College of Life Science and Technology, Beijing University of Chemical Technology, Beijing, 100029, People's Republic of China; c Qingyuan Innovation Laboratory, Quanzhou, 362801, People's Republic of China

**Keywords:** Fumarate, Cell-free, Multi-enzyme catalysis, Acetate, Glyoxylate

## Abstract

Fumarate is a value-added chemical that is widely used in food, medicine, material, and agriculture industries. With the rising attention to the demand for fumarate and sustainable development, many novel alternative ways that can replace the traditional petrochemical routes emerged. The *in vitro* cell-free multi-enzyme catalysis is an effective method to produce high value chemicals. In this study, a multi-enzyme catalytic pathway comprising three enzymes for fumarate production from low-cost substrates acetate and glyoxylate was designed. The acetyl-CoA synthase, malate synthase, and fumarase from *Escherichia coli* were selected and the coenzyme A achieved recyclable. The enzymatic properties and optimization of reaction system were investigated, reaching a fumarate yield of 0.34 mM with a conversion rate of 34% after 20 h of reaction. We proposed and realized the conversion of acetate and glyoxylate to fumarate *in vitro* using a cell-free multi-enzyme catalytic system, thus providing an alternative approach for the production of fumarate.

## 1 Introduction

Fumarate, a four-carbon (C4) dicarboxylic acid, is widely used for producing paper resins, plasticizers, food, and medicine additives due to its one carbon-carbon double bond and two carboxylic acid groups [1,2]. Recently, growing demand for this value-added product has emerged, resulting in more and more scientific researches being attracted to fumarate production through various approaches aimed to increase the production capacity [[3], [4], [5]]. At present, there are three main methods including petrochemical, enzymatic, and microbial routes for the synthesis of fumarate. Petrochemical routes are now the most common method to produce fumarate, and it can be achieved through the isomerization of maleic acid [6]. Recently, isomerization of maleic acid in water without catalyst was investigated, which reached a maximum yield of 86% fumarate from 80% maleic acid [7]. Another way to produce fumarate is the enzymatic route. Because of the restriction of reaction equilibrium and formation of byproducts during the chemical processes, catalysis approaches using enzymes from microorganisms provide an alternative method. The core idea is to utilize maleate *cis-trans* isomerase to isomerize maleic acid into fumarate. Some microorganisms such as *Pseudomonas* species and *Alcaligenes faecalis* are able to synthesize maleate *cis-trans* isomerase with different activities [2,8]. Nevertheless, the poor thermal stability of this isomerase limited its application, thus certain high activity and thermos-stable maleate isomerase candidates were excavated [9,10].

To meet the needs of sustainable development and environmental protection, the utilization of microbial fermentation by various strains such as *Rhizopus oryzae*, *Escherichia coli*, *Saccharomyces cerevisiae*, *Torulopsis glabrata* and their mutants for fumarate production using renewable bioresources has been widely studied [11]. Many researches have considered that *Rhizopus* species are the best fumarate producers. For example, a previous study obtained the highest titer of 121 g/L of fumarate in stirred tank using wild-type *R. nigricans* [12]. Another study also performed in stirred tank fermenter, and 56.2 g/L of fumarate with 0.54 g/g yield on glucose was achieved using a two-stage dissolved oxygen control strategy [13]. Even though, the requirement for rich nutrients of *Rhizopus* species is a main shortcoming as it affects the economic competitiveness. Therefore, model microorganisms such as *E. coli* and *S. cerevisiae* were metabolically engineered to achieve fumarate accumulation. For example, *E. coli* was engineered to redistribute the carbon flux from glycolysis to Krebs cycle and block the byproducts formation, yielding 1.7 g/L fumarate using glucose as carbon source [14]. Another engineered *E. coli* produced 41.5 g/L fumarate from glycerol with an overall productivity of 0.51 g/L/h in an 82-h fed-batch fermentation. The modified strain could significantly reduce acetate accumulation and increase fumarate production, which was the highest record for fumarate production by *E. coli* to date [15]. Moreover, a systematic modular pathway engineering was implemented in *S. cerevisiae*. After operating three modules including reduction, oxidation, and byproduct module, a fairly high titer of 33.13 g/L was obtained in shake flask cultivations [16].

The technology of cell-free multi-enzyme synthesis has been investigated and applied for a long time since the 1960's [17,18]. It allows the assembly of a number of purified enzymes and coenzymes for the production of biocommodities in an *in vitro* system [19]. Currently, the cell-free synthetic technology is becoming a promising approach that possessed potential to replace the traditional chemical methods with the rapid advance and development of synthetic biology [20]. Generally, microbial fermentation remains restricted to the complicated intracellular metabolic activities and physiological limits [21]. The metabolic fluxes were often channeled into the non-targeted products due to the not fully understood pathways, which resulted in waste of energy and then caused low conversion efficiencies and yields. Therefore, the cell-free multi-enzyme synthesis aroused great interests due to its concise pathways and effective energy supply [22]. Now there are more and more studies that focused on the production of biofuels or high-value chemicals using cell-free synthetic systems, such as hydrogen [23], 2,3-butanediol [24], l-lactate [25], acetoin [26], and fatty acid [27]. Recently, a cell-free system harboring chemical-biochemical hybrid pathways were established to synthesize starch using carbon dioxide (CO_2_) and hydrogen as substrates, indicating the possibility of novel industrial production of starch [28].

Based on the unique advantages of *in vitro* cell-free catalysis, we tried to develop an alternative way to produce fumarate. The pathway involves only three enzymes, acetyl coenzyme A synthase, malate synthase, and fumarase, which can be used to produce fumarate in a cell-free multi-enzyme catalytic system using acetate and glyoxylate as the substrates. The process allows for the recycling of coenzyme A (CoA). The concentration of fumarate in the system could be increased to a considerable degree after optimizing the reaction conditions.

## 2 Materials and methods

### 2.1 Strains and plasmids

*Escherichia coli* DH5α was employed as the host for plasmid construction and preservation. *E. coli* BL21 (DE3) was used as the host strain for the expression of the his-tagged enzymes. The genes involved in fumarate synthesis pathway were cloned from the genomic DNA of *E. coli* DH5α using the corresponding primers. Plasmid pET28a was employed as the vector for gene expression. The plasmids and primers used in this study are listed in Table 1 and Table S1, respectively.

**Table 1:** Plasmids used in this study.

Name	Description	Reference
pET28a	Expression vector, T7 promoter	Novagen
pET28a-acs	pET28a derived, harboring *acs* gene of *E. coli*	This study
pET28a-ackA	pET28a derived, harboring *ackA* gene of *E. coli*	This study
pET28a-pta	pET28a derived, harboring *pta* gene of *E. coli*	This study
pET28a-aceB	pET28a derived, harboring *aceB* gene of *E. coli*	This study
pET28a-glcB	pET28a derived, harboring *glcB* gene of *E. coli*	This study
pET28a-fumA	pET28a derived, harboring *fumA* gene of *E. coli*	This study
pET28a-fumB	pET28a derived, harboring *fumB* gene of *E. coli*	This study
pET28a-fumC	pET28a derived, harboring *fumC* gene of *E. coli*	This study

For the expression of acetyl-CoA synthesis enzymes, the PCR products of gene *acs* and *ackA* were purified and digested with *Bam*HI/*Hin*dIII, and the PCR product of gene *pta* was purified and digested with *Eco*RI/*Hin*dIII. Then, the gene fragments were all ligated to pET28a cut with the same enzymes to yield pET28a-acs, pET28a-ackA, and pET28a-pta, respectively. Similarly, the PCR products of genes *glcB* and *aceB* were purified and digested with *Bam*HI/*Hin*dIII and *Eco*RI/*Hin*dIII, respectively. The PCR products of genes *fumA*, *fumB*, and *fumC* were purified and digested with *Bam*HI/*Eco*RI, *Bam*HI/*Eco*RI, and *Bam*HI/*Sca*I, respectively. Then the digested DNA fragments were inserted into the corresponding restriction sites of pET28a to yield malate synthase expression vectors (pET28a-glcB and pET28a-aceB) and fumarate synthase expression vectors (pET28a-fumA, pET28a-fumB, and pET28a-fumC), respectively.

### 2.2 Enzyme expression and purification

*E. coli* BL21(DE3) recombinant carrying the corresponding expression plasmid was grown in LB medium containing 50 μg/mL kanamycin at 37 °C and 200 rpm in a rotary shaker. IPTG was added to a final concentration of 1 mM when OD_600_ of the culture reached about 0.6, then the culture was further grown at 16 °C for 16 h. The cells were harvested by centrifugation at 4 °C, and the pellets were washed once using 10 mM Tris-HCl buffer (pH 7.5), then suspended with the same buffer for ultrasonication to prepare the crude cell lysate. Subsequently, the cell lysate was centrifuged at 10000 g for 10 min at 4 °C. The supernatant contained enzyme extract was further purified by loading to Ni-NTA His-Bind column. The purified protein was subject to SDS-PAGE to investigate the enzyme expression and purity. Protein concentration was analyzed by the Bradford assay.

### 2.3 Enzyme activity assays

The activity of acetyl-CoA synthase was assayed by measuring the increase of NADH at 340 nm as the methods reported previously. The reaction mixture (1 mL) contained 100 μL purified enzyme, 100 mM Tris-HCl buffer (pH 8.0), 10 mM MgCl_2_, 1 mM NAD^+^, 0.2 mM CoA, 8 mM ATP, 10 mM malate, 3 U malate dehydrogenase, 0.4 U citrate synthase, and 100 mM sodium acetate [29]. The activity of phosphate acetyltransferase was determined by measuring the change of value before and after the addition of acetyl-CoA at 412 nm. The reaction mixture (1 mL) contained 100 μL purified enzyme, 100 mM phosphate buffer (pH 7.4), 0.2 mM acetyl-CoA, 0.1 mM 5,5′-dithiobis(2-nitrobenzoic acid) (DTNB) [30]. The activity of acetate kinase was assayed by measuring the reduction of NADH at 340 nm, and the reaction mixture (1 mL) contained 100 μL purified enzyme, 70 mM Tris-HCl buffer (pH 7.6), 50 mM MgCl_2_, 0.3 mM NADH, 3 mM ATP, 3 mM phosphoenolpyruvate, 7 U pyruvate kinase, 10 U lactate dehydrogenase, and 340 mM sodium acetate [30]. The activity of malate synthase was determined by measuring the change in absorbance before and after the addition of DTNB at 412 nm. The reaction mixture (1 mL) contained 100 μL purified enzyme, 25 mM Tris-HCl buffer (pH 8.0), 100 mM MgCl_2_, 0.25 mM acetyl-CoA, 1 mM glyoxylate, and 0.2 mM DTNB [31]. The activity of fumarase was assayed spectrophotometrically by measuring the formation of fumarate at 250 nm in 100 mM phosphate buffer (pH 7.5) containing 50 mM malate [32]. One unit of enzyme activity was defined as the amount of enzyme that released 1 μmol of product per minute.

### 2.4 Fumarate synthesis and assay

The cell-free multi-enzyme catalytic reaction for fumarate synthesis was conducted in a 1.0-mL system containing 50 mM Tris-HCl buffer (pH 7.5), 10 mM MgCl_2_, appropriate substrates (1 mM acetate, 1 mM glyoxylate, 0.2 mM CoA, 1 mM ATP), and the corresponding enzymes at 37 °C. The buffer and substrates were first put into a vessel, then the enzymes were added and mixed quickly with the substrates to start the reaction. The initial amounts for each enzyme were used as follows, 0.01 U/mL Acs, 0.01 U/mL AckA, 0.002 U/mL Pta, 0.01 U/mL GlcB, 0.2 U/mL FumC. To increase the yield of fumarate in the system, the amount of each enzyme, CoA, ATP, and MgCl_2_ were optimized sequentially. The loading amount of one specific enzyme or substrate was altered each time, while the other enzymes and substrates were added at the same amounts as described in the initial condition. Once a specific optimal value was determined, it was used in the subsequent experiments. The concentration of acetate, glyoxylate, malate, and fumarate were determined by high performance liquid chromatography (HPLC) using an ion exchange column (Aminex HPX-87H, 7.8 × 300 mm, BioRad) and a UV detector. The column was kept at 55 °C and was eluted using a mobile phase comprising 5 mM H_2_SO_4_ at a flow rate of 0.6 ml/min.

## 3 Results and discussion

### 3.1 Design of the pathway for fumarate production

**Fig. 1:**
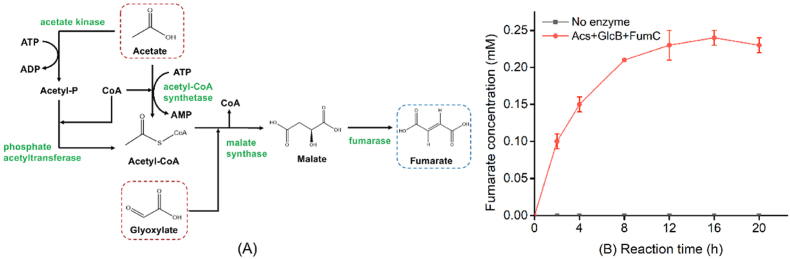
(A) Schematic illustration of the *in vitro* multi-enzyme catalytic pathway that converts acetate and glyoxylate to fumarate. (B) Fumarate production under initial conditions. The reaction mixture contained 0.01 U/mL Acs, 0.01 U/mL GlcB, 0.2 U/mL FumC, 10 mM MgCl_2_, 0.2 mM CoA, 1 mM ATP, 1 mM acetate, and 1 mM glyoxylate.

An *in vitro* multi-enzyme catalytic pathway that converts acetate and glyoxylate to fumarate was rationally designed. As shown in Fig. 1A, this pathway can be decomposed into two modules, acetyl-CoA synthesis module and fumarate synthesis module. The first module is responsible for the formation of acetyl-CoA from acetate, and there are two routes to achieve this process. For one thing, acetate can be phosphorylated by acetate kinase (AckA) to generate acetyl phosphate (acetyl-P), which then be converted to acetyl-CoA by phosphate acetyltransferase (Pta). For another, one molecule of acetate is directly transformed into acetyl-CoA by combing with CoA in the same proportion by acetyl-CoA synthetase (Acs). Both of the pathways above had been implemented for further study. The second module catalyzes the conversion of acetyl-CoA and glyoxylate to produce fumarate. Malate synthase and fumarase are involved in it with releasing of CoA. Importantly, the condensation of acetyl-CoA and glyoxylate by malate synthase prefers to generate malate, since the split of malate into glyoxylate and acetyl-CoA is thermodynamically unfavorable due to the ΔrG' (44.4 kJ/mol) [33]. There was also more than one enzyme to be chosen in the two-step reactions. Two malate synthase isoenzymes (AceB and GlcB) and three fumarase isoenzymes (FumA, FumB, and FumC) were selected for further comparison [34,35]. As a result, one molecule of the four-carbon compound fumarate can be synthesized from the two-carbon (C2) building blocks acetate and glyoxylate.

### 3.2 Enzyme characterization and selection

The plasmids carrying genes encoding for the required enzymes were transformed into *E. coli* BL21(DE3) to construct the recombinant strains. The expressed enzymes with 6 × His tags were purified and determined by SDS-PAGE. As shown in Figs. S1, S2, and S3, the bands of the crude and purified enzyme solution were clearly indicated. It was suggested that all enzymes were expressed in large quantities and the protein purity was high. The molecule weights of all enzymes were in agreement with the theoretical molecule weight values. Subsequently, the catalytic activities of the enzymes were determined and the results were shown in Table 2. The isoenzymes including malate synthase and fumarase were compared. The enzyme activity values of AceB and GlcB were 0.22 and 0.30 U, respectively. The enzyme activity values of FumA, FumB, and FumC were 10.04, 3.37, and 11.47 U, respectively. Previously it was reported that FumA and FumB were thermolabile and iron-dependent Class I fumarase [34]. Thus, GlcB and FumC were then selected for the following catalysis studies according to the results of enzyme expression and activities.

**Table 2:** The activities of enzymes involved in multi-enzyme catalysis.

Enzyme	Abbreviation	Activity (U)	Specific activity (U/mg)
Acetyl-CoA synthetase	Acs	0.77 ± 0.14	0.17 ± 0.03
Acetate kinase	AckA	1.43 ± 0.15	0.63 ± 0.07
Phosphate acetyltransferase	Pta	0.14 ± 0.02	0.05 ± 0.01
Malate synthase A	AceB	0.22 ± 0.06	0.05 ± 0.01
Malate synthase G	GlcB	0.30 ± 0.01	0.07 ± 0.01
Fumarase A	FumA	10.04 ± 0.25	4.13 ± 0.10
Fumarase B	FumB	3.37 ± 0.50	1.52 ± 0.23
Fumarase C	FumC	11.47 ± 0.25	3.71 ± 0.08

### 3.3 Fumarate production under initial condition

The synthesis of fumarate was first conducted by an initial cell-free system containing 0.01 U/mL Acs, 0.01 U/mL GlcB, and 0.2 U/mL FumC. The optimal pH, one of the important factors affecting enzyme activity, was set at 7.4 in 50 mM Tris-HCl buffer in the 1.0-mL reaction system containing 10 mM MgCl_2_, 0.2 mM CoA, and 1 mM ATP. The substrates including acetate and glyoxylate were added at 1 mM due to their toxicity and corrosiveness. As shown in Fig. 1B, the concentration of fumarate increased continuously with the extension of reaction time, and reached 0.23 mM after 12 h. There was no obvious increase in the next few hours. It was observed that the accumulation of fumarate reached a maximum value of 0.24 mM with a conversion rate of 24% at 16 h, which was only an increase of 0.01 mM over the last 4 h.

The reaction catalyzed by fumarase is reversible. Although there should be malate existed in the reaction mixture, the malate peak could not be detected through HPLC analysis due to its low concentration. In addition, the dehydration of malate to produce fumarate in aqueous condition is entropy unfavorable. Previously, the equilibrium constant of conversion of malate to fumarate mediated by fumarase was reported to be 1:4.2 (fumarate to malate) [36]. To overcome the limitation, ethylene glycol has been applied as a hydrophilic solvent to improve the conversion yield using a thermostable fumarase from *Thermus thermophilus* HB8 [9]. Besides, the enzymes in current reaction system were in free state, and their catalytic activity may be affected by the stability. Multienzyme co-immobilization technologies could be developed using carriers such as nanofibers, membrane, and biomimetic materials to improve the stability and reusability, which may help to further improve the efficiency of fumarate accumulation [37].

### 3.4 Optimization of reaction conditions for improved production of fumarate

**Fig. 2:**
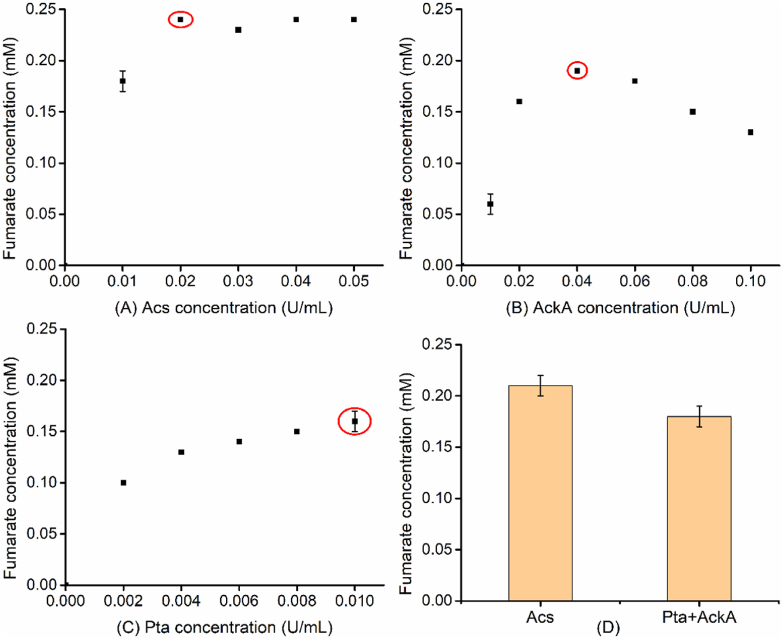
Optimization of acetyl-CoA synthesis-related enzymes for higher production of fumarate. (A), Effect of Acs concentration from 0 to 0.05 U/mL on fumarate production. (B), Effect of AckA concentration from 0 to 0.10 U/mL on fumarate production. (C), Effect of Pta concentration from 0 to 0.010 U/mL on fumarate production. (D) The comparison of two acetyl-CoA synthesis pathways for fumarate production. The points in the red circle indicate the optimal condition for each parameter. The other components in the reaction mixture were as follows, 0.01 U/mL GlcB, 0.2 U/mL FumC, 10 mM MgCl_2_, 0.2 mM CoA, 1 mM ATP, 1 mM acetate, and 1 mM glyoxylate. The reaction time was 16 h.

Next, the optimization of enzyme loading amounts and other components was implemented. A series of acetyl-CoA synthesis-related enzymes (Acs, AckA, and Pta) were first optimized (Fig. 2). Firstly, Acs was set at 0, 0.01, 0.02, 0.03, 0.04, and 0.05 U/mL in six concentration gradients. When the amount of Acs was 0.02 U/mL, fumarate concentration reached a maximum and no longer increased with extra amounts of enzyme. In terms of AckA, there was an increasing trend in fumarate formation followed by a decreasing trend when AckA was used from 0.01 to 0.10 U/mL. It was found that 0.04 U/mL was the optimal loading amount, which maximized the accumulation of fumarate. For the optimization of Pta, there was a flat incremental trend with the increase of enzyme loading amount, and 0.01 U/mL of Pta was employed as the optimal loading amount. Subsequently, the comparison of two acetyl-CoA synthesis pathways was investigated. As shown in Fig. 2D, the final concentration of fumarate catalyzed by Acs reached 0.21 mM, which was significantly higher than that catalyzed by the Pta-AckA reaction system. Therefore, 0.02 U/mL of Acs was chosen for the synthesis of acetyl-CoA in further optimization process.

**Fig. 3:**
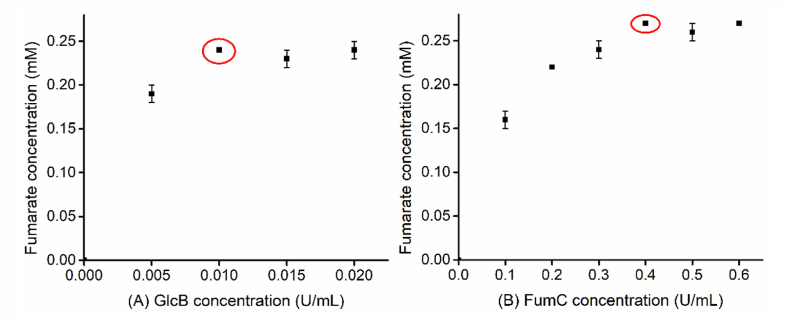
Optimization of fumarate synthesis-related enzymes for higher production of fumarate. (A), Effect of GlcB concentration from 0 to 0.020 U/mL on fumarate production. (B), Effect of FumC concentration from 0 to 0.6 U/mL on fumarate production. The points in the red circle indicate the optimal condition for each parameter. The other components in the reaction mixture were as follows, 0.02 U/mL Acs, 10 mM MgCl_2_, 0.2 mM CoA, 1 mM ATP, 1 mM acetate, and 1 mM glyoxylate. The reaction time was 16 h.

We next optimized the enzymes involved in the second module, malate synthase and fumarase, which convert acetyl-CoA and glyoxylate to fumarate. Different concentration gradients of 0–0.02 U/mL were implemented for GlcB, and 0–0.6 U/mL were applied for FumC. As shown in Fig. 3, it was demonstrated that the optimal enzyme loading value of GlcB and FumC were 0.01 and 0.4 U/mL, respectively. Since the two enzymes were optimized sequentially, as well as the reversibility of the second reaction, optimization for GlcB didn't show a clear change in yield. However, obvious change was observed in the optimization for FumC as a result of more materials proceeding in the positive direction.

**Fig. 4:**
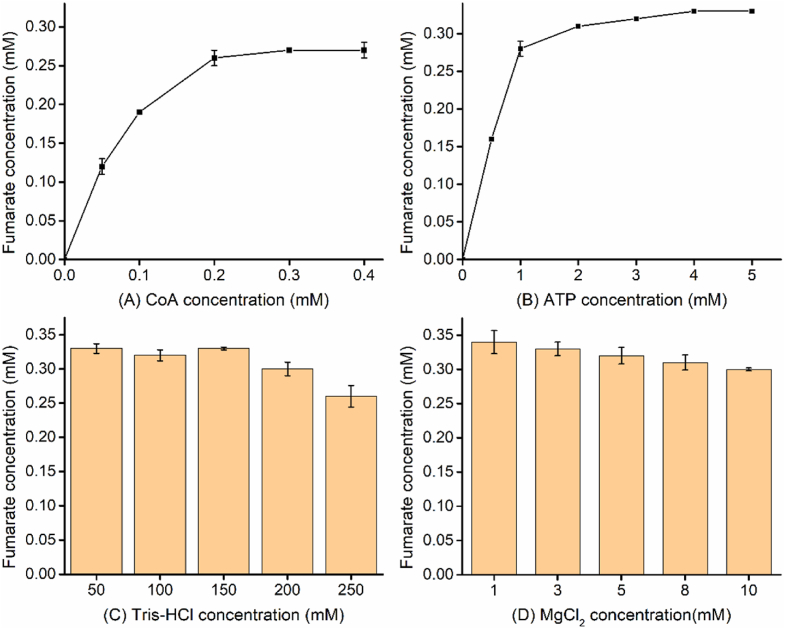
Optimization of other components for higher production of fumarate. (A), Effect of CoA concentration from 0 to 0.4 mM on fumarate production. (B) Effect, of ATP concentration from 0 to 5 mM on fumarate production. (C), Effect of Tris-HCl concentration from 50 to 250 mM on fumarate production. (D), Effect of MgCl_2_ concentration from 1 to 10 mM on fumarate production. The reaction mixture contained 0.02 U/mL Acs, 0.01 U/mL GlcB, and 0.4 U/mL FumC. Both acetate and glyoxylate were added at the concentration of 1 mM.

After the enzyme loading amounts were all determined, the concentrations of other components including CoA, ATP, Tris-HCl, and MgCl_2_ were also optimized (Fig. 4). It was found that linear increase of fumarate followed by levelling off when the concentration of CoA was applied from 0 to 0.4 mM with five different gradients. Next, the concentration of ATP was adjusted and it was found that the accumulation of fumarate rapidly climbed to about 0.3 mM when ATP concentration increased from 0 to 2 mM. The fumarate production levelled off with more than 2 mM of ATP added. Therefore, 0.2 mM of CoA and 2 mM of ATP as the inflection points were used as the optimal concentrations, respectively. Subsequently, the optimal concentration of Tris-HCl buffer was determined (Fig. 4C). It was observed that there was no significant impact on the accumulation of fumarate with Tris-HCl ranging from 50 to 150 mM. While Tris-HCl buffer above 200 mM resulted in decreased fumarate formation. Finally, we investigated the effects of MgCl_2_ addition on fumarate synthesis, and the minimal concentration (1 mM) was selected due to the gradual reduction of fumarate with the increased MgCl_2_ concentration (Fig. 4D).

### 3.5 Fumarate production under optimized conditions

**Fig. 5:**
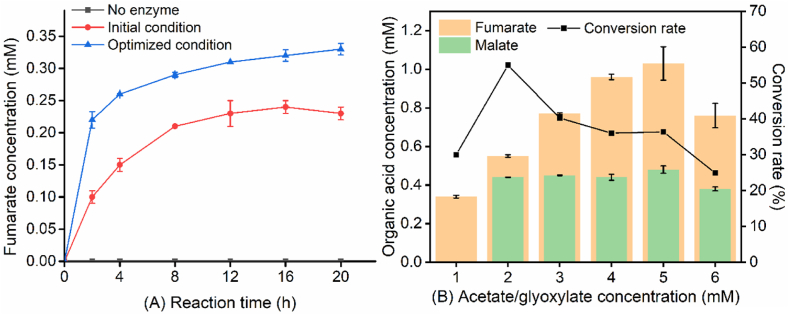
Fumarate production under optimized conditions. (A), Comparison of initial and optimized conditions for fumarate production. (B), Effect of substrate concentration on the conversion rate.

Finally, the cell-free multi-enzyme catalysis reaction for fumarate synthesis was carried out under the optimized condition, in which the reaction mixture contained 0.02 U/mL Acs, 0.01 U/mL GlcB, 0.4 U/mL FumC, 1 mM MgCl_2_, 0.2 mM CoA, 2 mM ATP, 1 mM acetate, and 1 mM glyoxylate (Fig. 5A). The yield of fumarate increased significantly during the first 2 h of reaction, and levelled off after 12 h. It was found that the concentration of fumarate reached the highest value of 0.34 mM at 20 h, which was about 42% higher than that under initial conditions. The conversion rate of fumarate from the substrates was increased from 23% to 34%. There was trace amount of malate detected from HPLC analysis, yet the detailed value cannot be determined due to the low amount.

Compared to the initial reaction conditions, the main changes in the optimized conditions were an increase in the amount of Acs/FumC and a significant decrease in the concentration of MgCl_2_ (from 10 to 1 mM). There was more malate observed owing to the increased addition of fumarate synthase in the system. FumC used in this study differs from FumA and FumB that it is a class II enzyme which does not require iron for its activity [38]. Besides, large changes in fumarate concentration with the changes in the amount of FumC, which might be a rate-limiting enzyme. The fumarase cannot efficiently convert malate to fumarate because the dehydration of malate was unfavorable in aqueous condition. To deal with this problem, rational design or site-directed saturated mutagenesis based on machine learning could be used to enhance enzyme activity towards malate to fumarate [39,40].

To study the effect of substrate concentration on the conversion rate, acetate and glyoxylate with different concentrations in the cell-free system were investigated under optimized conditions. As shown in Fig. 5B, the production of fumarate was increased gradually with the increase of substrate concentration. However, the accumulation of malate in the cell-free system showed a significant increase to about 0.4 mM. It was observed that fumarate reached the highest conversion rate of 55% at 2 mM of acetate/glyoxylate and the highest yield of 1.03 mM at 5 mM of acetate/glyoxylate. When 6 mM of acetate/glyoxylate was applied, both fumarate and malate amounts showed a decreasing trend, probably caused by the substrate inhibition effect [41]. Further development of highly efficient catalysts and biological enzymes would help to improve the productiveness of the artificial cell-free catalytic system.

In the current cell-free catalytic system for fumarate production, ATP was required for the activation of acetate and further introduction of an ATP regeneration system would be a crucial approach to decrease the feedstock cost. Polyphosphate kinase (PPK) can phosphorylate AMP or ADP to synthesize ATP using inorganic polyphosphate (polyP_n_) as the phosphate donor. The application of PPK/polyP_n_ as an efficient ATP regeneration system has been demonstrated in the enzymatic synthesis of various biomolecules [42,43]. In terms of the substrates, acetate can be efficiently produced from CO_2_ through the electrochemical reduction, and also the acetogens such as *Acetobacterium woodii* or *Clostridium ljungdahlii* accumulated acetate as a major product by feeding gas mixture of H_2_ and CO_2_ [44,45]. Recently, the oxygenase function of Rubisco was developed to convert CO_2_ into glycolate, and the engineered cyanobacteria accumulated 2.8 g/L glycolate from CO_2_ [46,47]. Glycolate is easily converted to glyoxylate by glycolate oxidase [47]. Further combination of the above progress and multi-enzyme catalytic system of this study would provide a possibility of fumarate synthesis using CO_2_ as the substrate, which is an attractive route to decrease CO_2_ emission and convert it into value-added chemicals.

## 4 Conclusions

In this study, the successful production of fumarate from acetate and glyoxylate using a cell-free multi-enzyme catalytic system was established for the first time, thereby achieving *in vitro* conversion of C2 compounds to C4 organic acid. The pathway involves only three enzymes, acetyl-CoA synthase, malate synthase, and fumarase. The enzymatic properties and optimization of reaction system were investigated. The yield of fumarate reached 0.34 mM with a conversion rate of 34% was achieved after 20 h of reaction. Although the concentration is much lower than that produced by fermentation, further research including the rational design of enzymes and appropriate immobilization strategies would be benefit for improved catalytic activity and fumarate production. This study provides an alternative approach for fumarate synthesis and may promote the development of cell-free multi-enzyme biosystems for biomanufacturing.

## Declaration of competing interest

The authors declare that they have no known competing financial interests or person relationships that could have appeared to influence the work reported in this paper.

## CRediT authorship contribution statement

**Congli Hou:** Investigation, Methodology, Visualization, Writing – original draft. **Linyue Tian:** Investigation, Visualization, Writing – original draft. **Guoli Lian:** Investigation, Validation. **Li-Hai Fan:** Management and, Supervision, Writing – review & editing, Funding acquisition. **Zheng-Jun Li:** Conceptualization, Data curation, Management and, Supervision, Writing – review & editing, Funding acquisition.
