# Supplementary material for: Conversion of acetate and glyoxylate to fumarate by a cell-free synthetic enzymatic biosystem
Source: Synth Syst Biotechnol. 2023 Mar 16;8(2):235–41. doi: 10.1016/j.synbio.2023.03.004 (PMC10033897; doi:10.1016/j.synbio.2023.03.004)
Supplement: Multimedia component 1 [file mmc1.docx]

**Supporting information**


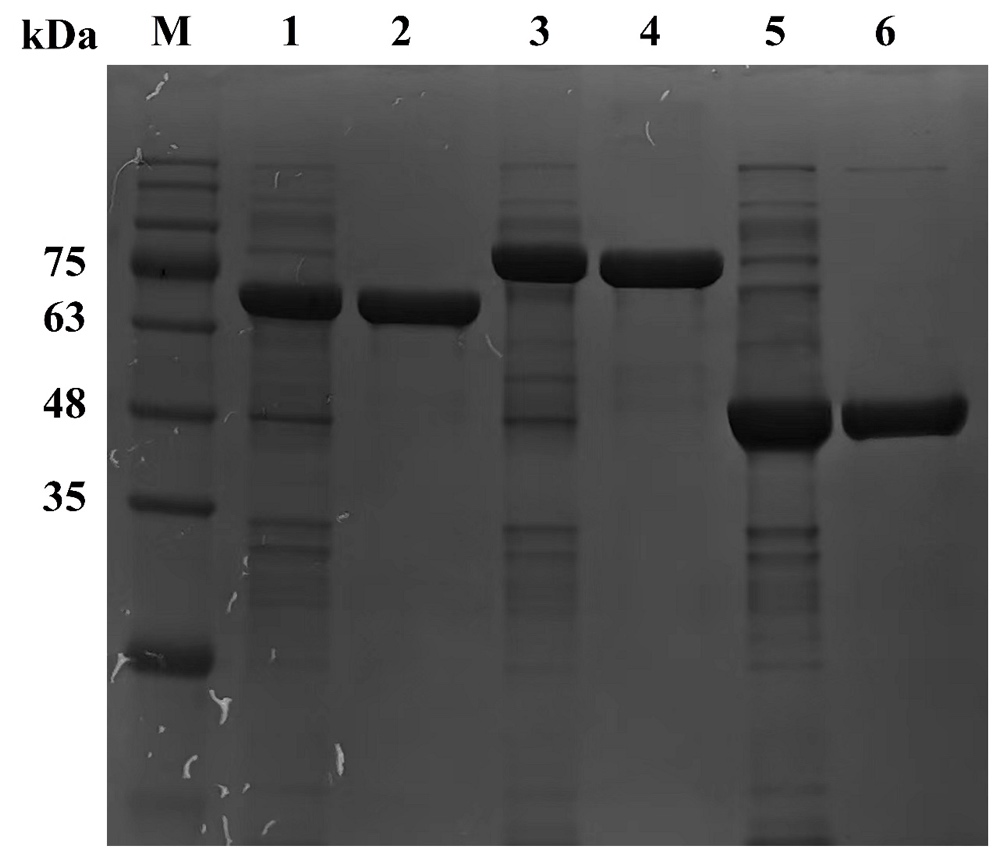
Fig. S1 SDS-PAGE analysis of Acs, Pta, and AckA.

Lanes 1, 3, and 5 refer to the crude enzyme solution of *E. coli* strains overexpressing Acs, Pta, and AckA. Lanes 2, 4, and 6 refer to the purified Acs, Pta, and AckA. Lane M, protein marker. The molecular weights of Acs, Pta, and AckA were 63, 70, 40 kDa, respectively.


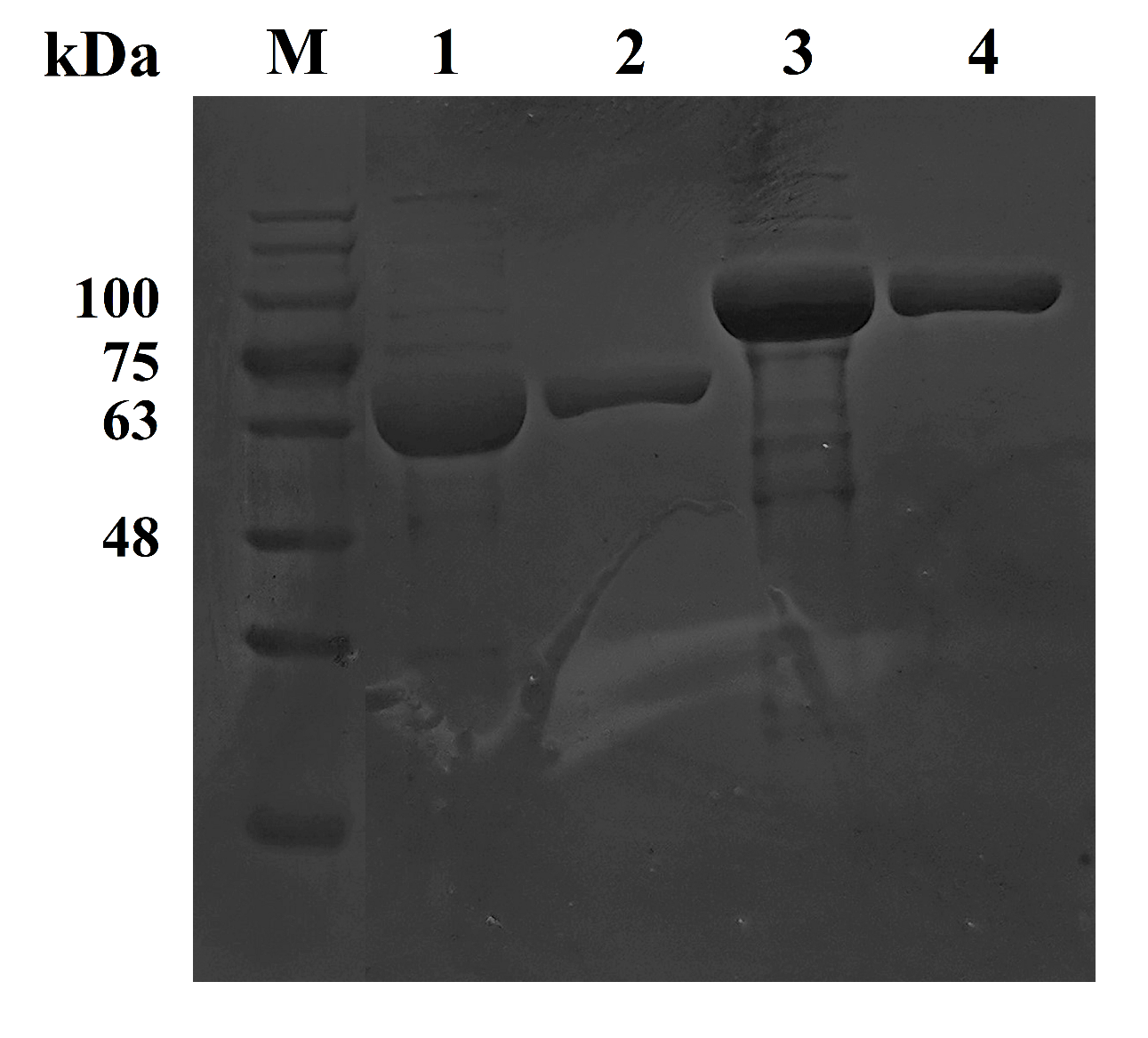
Fig. S2 SDS-PAGE analysis of AceB and GlcB.

Lanes 1 and 3 refer to the crude enzyme solution of *E. coli* strains overexpressing AceB and GlcB. Lanes 2 and 4 refer to the purified AceB and GlcB. Lane M, protein marker. The molecular weights of AceB and GlcB were 53 and 90 kDa, respectively.


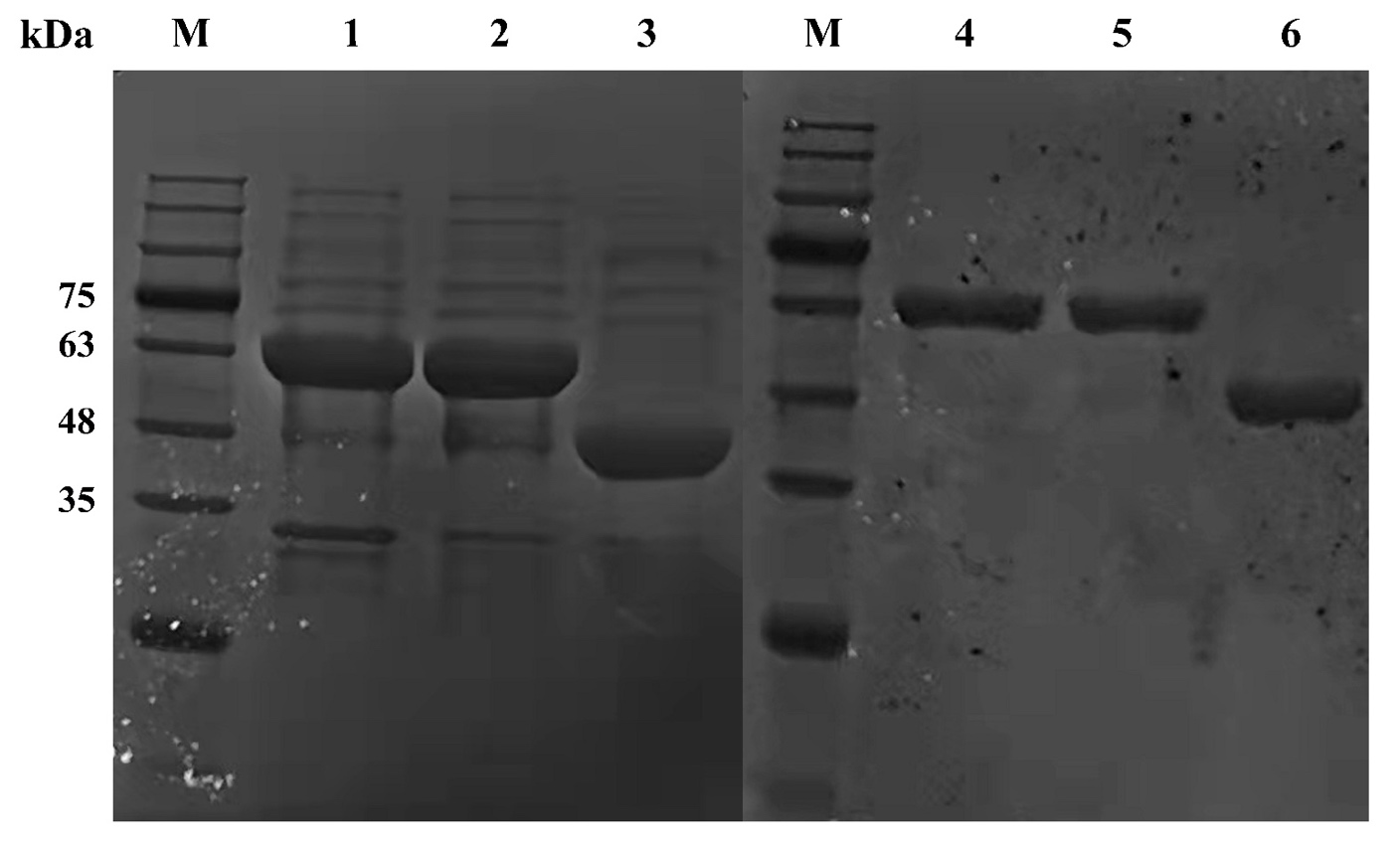
Fig. S3 SDS-PAGE analysis of FumA, FumB, and FumC.

Lanes 1, 2, and 3 refer to the crude enzyme solution of *E. coli* strains overexpressing FumA, FumB, and FumC. Lanes 4, 5, and 6 refer to the purified FumA, FumB, and FumC. Lane M, protein marker. The molecular weights of FumA, FumB, and FumC were 60, 60, 48 kDa, respectively.

Table S1 Primers used in this study.

| Name | Primer sequences (5’-3’) |
| --- | --- |
| acs-F | AAAGGATCCATGAGCCAAATTCACAAACACACC |
| acs-R | AAAAAGCTTTTACGATGGCATCGCGATAGCCT |
| ackA-F | AAAGGATCCATGTCGAGTAAGTTAGTACTGGTTCTG |
| ackA-R | AAAAAGCTTTCAGGCAGTCAGGCGGCTC |
| pta-F | AAAGAATTCGTGTCCCGTATTATTATGCTGATCCCTACC |
| pta-R | AAAAAGCTTTTACTGCTGCTGTGCAGACTGAA |
| aceB-F | AAAGAATTCATGACTGAACAGGCAACAACAACCGA |
| aceB-R | AAAAAGCTTTTACGCTAACAGGCGGTAGCCT |
| glcB-F | AAAGGATCCATGAGTCAAACCATAACCCAGAGCCGT |
| glcB-R | AAAAAGCTTTTAATGACTTTCTTTTTCGCGTAAACGCCAGG |
| fumA-F | AAAGGATCCATGTCAAACAAACCCTTTCATTATCAGG |
| fumA-R | AAAGAATTCTTATTTCACACAGCGGGTGCATT |
| fumB-F | AAAGGATCCATGTCAAACAAACCCTTTATCTACCAGGC |
| fumB-R | AAAGAATTCTTACTTAGTGCAGTTCGCGCACTGTTTGT |
